# Domestically Acquired NDM-1–Producing *Pseudomonas aeruginosa*, Southern California, USA, 2023

**DOI:** 10.3201/eid2911.230646

**Published:** 2023-11

**Authors:** Hannah K. Gray, Omer E. Beaird, Ethan A. Smith, Joanna M. Schaenman, Shangxin Yang

**Affiliations:** University of California, Los Angeles, California, USA

**Keywords:** Pseudomonas aeruginosa, bacteria, antimicrobial resistance, healthcare-associated infections, California, United States

## Abstract

We describe a case of New Delhi metallo-β-lactamase 1–producing carbapenem-resistant *Pseudomonas aeruginosa* (CRPA) in a transplant patient with multiple hospitalizations in California, USA. Whole-genome sequencing revealed the isolate was genetically distinctive, despite ≈95% similarity to other global strains. The patient’s lack of international travel suggests this CRPA was acquired domestically.

Carbapenem-resistant *Pseudomonas aeruginosa* (CRPA) is increasing worldwide and has up to 30% prevalence in US *P. aeruginosa* isolates (*1*). The Antimicrobial Resistance Laboratory Network reported 280 carbapenemase-producing CRPA in 2021, most commonly Verona integron metallo-β-lactamase (Centers for Disease Control and Prevention, https://arpsp.cdc.gov/profile/arln/crpa). New Delhi metallo-β-lactamase (NDM)–producing CRPA is prevalent in Eurasia and the Middle East. Sporadic NDM CRPA cases linked to international travel have been reported in the United States, the earliest of which was identified in Delaware in 2014 (*2*). We describe a case of NDM CRPA in southern California.

A previously healthy patient in his 50s was admitted to a hospital in Riverside County, California, in 2023 with cardiogenic shock secondary to new onset nonischemic cardiomyopathy. He was briefly admitted to the same hospital a few weeks earlier because of chest pain and dyspnea. He had no other healthcare exposures and had only traveled briefly to Hawaii earlier that year.

Shortly after the second admission, the patient experienced cardiac arrest, was cannulated for extracorporeal life support, and was transferred to Ronald Reagan UCLA Medical Center for heart transplant evaluation. Admission blood cultures grew *Bacillus cereus*, attributed to gastrointestinal translocation. The patient received vancomycin, resulting in bacteremia clearance, and empiric piperacillin/tazobactam for gram-negative bacteria coverage. Four days pretransplant, hypotension and leukocytosis worsened, and antimicrobial therapy was empirically changed to meropenem and amikacin. A urine culture sent during sepsis evaluation grew *P. aeruginosa*. On the basis of susceptibility testing results, cefiderocol was initiated; the patient received heart and kidney transplants the next day. Cefiderocol was continued for 6 days posttransplant; subsequent urine cultures were negative. *P. aeruginosa* was not isolated from other blood or respiratory cultures.

Initial susceptibility testing of the urine isolate revealed extensive resistance to carbapenems, aminoglycosides, fluoroquinolones, and cephalosporins, except cefiderocol (Appendix). Rapid Carba-5 (Hardy Diagnostics, https://hardydiagnostics.com) testing detected NDM. We performed whole-genome sequencing by using MiSeq (Illumina, https://www.illumina.com) and assembled reads de novo by using CLC Genomics Workbench (QIAGEN, https://www.qiagen.com). We submitted tentative assemblies to the Comprehensive Antibiotic Resistance Database Resistance Gene Identifier tool (https://card.mcmaster.ca/analyze/rgi) for resistance gene detection and verified results by using ResFinder (Center for Genomic Epidemiology, https://genomicepidemiology.org) (Table). The isolate contained 5 β-lactamase genes: class A extended-spectrum β-lactamase *bla*_PME-1_, class B carbapenemase *bla*_NDM-1_, class D oxacillinase *bla*_OXA-50_–type *bla*_OXA-488_ and *bla*_OXA-10_, and class C cephalosporinase *bla*_PDC-35_.

**Table:** Antimicrobial genetic markers detected in a case of domestically acquired NDM-1–producing *Pseudomonas aeruginosa*, southern California, USA, 2023

Resistance mechanism	Genes
Aminoglycoside modifying enzymes	*aac(6')-Ib9, ant(3”)-IIa, aph(3′)-IIb, aph(3′)-VIa*
β-lactamases	*bla*_NDM-1_, *bla*_OXA-10_, *bla*_OXA-488_, *bla*_PDC-35_, *bla*_PME-1_
Fluoroquinolone resistance determinant	*gyrA* (T83I), *parE* (S457R)
Chloramphenicol resistance determinant	*catB3*, *catB7*, *cmlA9*
Fosfomycin resistance determinant	*fosA*
Tetracycline resistance determinant	*tet(D)*
Sulfonamide resistance determinant	*sul1*

*NDM, New Delhi metallo-β-lactamase.

Multilocus sequence typing designated the isolate as sequence type (ST) 235, frequently associated with *bla*_NDM-1_, including in isolates from Serbia, France, and Italy (*3*,*4*). ST235 is considered a high-risk clone, notable for harboring multiple β-lactamases, causing invasive infections and high mortality rates (*5*). Although most metallo-β-lactamases detected in ST235 are imipenemase variants, NDM CRPA are globally disseminated and have been reported in Asia, Europe, the Middle East, and Africa (*5*). We also detected other resistance genes reported in ST235 strains, including aminoglycoside-modifying enzyme *aac(6')-Ib9* and chloramphenicol resistance genes *cmlA* and *catB7* (*6*). In an outbreak of NDM CRPA in Iran, 86.2% of isolates coharbored *bla*_OXA-10_ (*7*), which we also detected in the isolate in this case. Consistent with other *bla*_NDM-1_–positive isolates, *bla*_NDM-1_ in this isolate was flanked by IS91-type insertion sequences, indicating mobilizability (*8*). The isolate also exhibited intermediate susceptibility to colistin, sometimes used to treat carbapenemase-producing CRPA. The isolate lacked an *mcr* gene, indicating mutations in *pmrAB* could be responsible for this phenotype, consistent with other ST235 carbapenemase-producing CRPA (*9*).

We used CSIPhylogeny (Center for Genomic Epidemiology) to perform single-nucleotide polymorphism (SNP) analysis against other NDM-1–producing ST235 isolates, then CLC Bioinformatics Workbench (QIAGEN) to generate a phylogenetic tree. The isolate from this study exhibited highest homology (≈30 SNPs difference) with 2 non–NDM-producing ST235 isolates from Malaysia (Appendix). Among NDM-producing carbapenemase-producing CRPA, the isolate clustered with an NDM CRPA from Italy (147 SNPs distance), suggesting origin in Europe (Figure). Among US strains, the isolate was genetically distinct from all NDM CRPA strains in isolate banks (Appendix Table 3) and a travel-associated strain from Texas (>20,000 SNPs distance) (*10*). That finding, and our patient’s lack of international travel, suggest that a domestic NDM CRPA strain is circulating in southern California. The patient received care at multiple institutions, making the precise origin of this strain unknown. Before this isolate, the rate of NDM–producing organisms at our institution remained low, <3 carbapenem-resistant Enterobacterales isolated annually, with no NDM CRPA.

**Figure:**
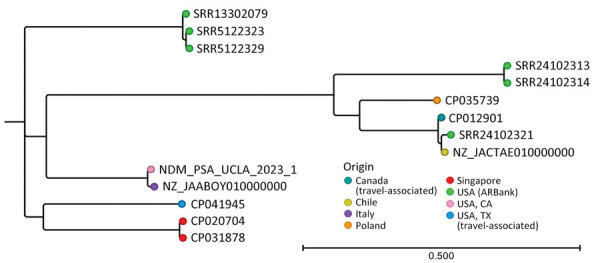
Phylogenetic tree for domestically acquired NDM-1–producing *Pseudomonas aeruginosa*, southern California, USA, 2023. Node colors indicate geographic location of organism isolation; the isolate described in this case report is designated as NDM_PSA_UCLA_2023_1. Accession numbers are provided for reference sequences. Scale bar indicates nucleotide substitutions per site. ARBank, CDC & FDA Antimicrobial Resistance (AR) Isolate Bank (https://www.cdc.gov/drugresistance/resistance-bank); NDM, New Delhi metallo-β-lactamase; UCLA, University of California Los Angeles.

The NDM CRPA isolate we report exhibited susceptibility to cefiderocol, which was used to clear the urinary tract infection. Upon phenotypic carbapenem resistance identification, cefiderocol susceptibility testing indicated sensitivity. The rapid availability of susceptibility testing results and preliminary testing performed within 24 hours after isolation were crucial for appropriate clinical management and antimicrobial drug choice, leading to safe heart transplantation and receipt of immunosuppression. Carbapenemase-producing bacteria should not disqualify a patient from transplantation. 

In conclusion, high mortality rates of ST235 NDM CRPA in invasive infection and a potential community spread in southern California warrant concern. The mobilization potential of *bla*_NDM-1_ remains unknown. Infection control measures and expanded surveillance efforts, including routine laboratory screening of all CRPA isolates via carbapenemase tests, could curb the spread of this high-risk genotype.

Appendix Additional information domestically acquired NDM-1–producing *Pseudomonas aeruginosa*, Southern California, USA, 2023.
